# Recent Advances in Graphene-Based Field-Effect Transistor Biosensors for Disease Biomarker Detection and Clinical Prospects

**DOI:** 10.3390/bios16040190

**Published:** 2026-03-26

**Authors:** Deeksha Nagpal, Anup Singh, John Link, Abijeet Singh Mehta, Ashok Kumar, Vinay Budhraja

**Affiliations:** 1 University Centre for Research and Development, Chandigarh University, Mohali 140413, India; 2 Department of Electrical Engineering, College of Engineering and Engineering Technology, Northern Illinois University, Dekalb, IL 60115, USA; 3 Department of Health and Biomedical Sciences, University of Texas Rio Grande Valley, Brownsville, TX 78521, USA; 4 Department of Applied Sciences, National Institute of Technical Teachers Training and Research, Chandigarh 160019, India

**Keywords:** graphene field-effect transistor, biomarker detection, clusterin, thrombin, estrogen receptor α, microRNAs, hepatocellular carcinoma, cytokine IL-6, prostate-specific antigen

## Abstract

Field-effect transistor (FET) biosensors using graphene have become one of the most promising biosensing platforms for the early diagnosis of diseases with features such as high sensitivity, label-free detection and application compatibility with point-of-care systems. Herein, we critically discuss recent advances in graphene FET (GFET) biosensor development toward clinically relevant biomarkers associated with representative diseases including cancer, neurodegenerative disease, infectious disease, and inflammatory conditions. Recent progress was reviewed to evaluate GFET architectures, surface functionalization methods, and detection quality. The biomarkers explored were clusterin in Alzheimer’s disease, thrombin in coagulopathy, estrogen receptor α (ER-α) in breast cancer, Carcinoembryonic antigen in lung cancer, microRNAs for malignant tumors, exosomes derived from HepG2 for the hepatocellular carcinoma (HCC) cell line, interleukin-6 (IL-6) for chronic obstructive pulmonary disease (COPD), Polyclonal antibodies and antigens (P24) for HIV and prostate-specific antigen for prostate cancer. The developed devices demonstrate ultralow detection limits at femtomolar to attomolar concentrations with the aid of designed antibodies, aptamers and nanomaterials. Herein, this review presents the sensing mechanisms and biomedical application of various GFET platforms, focusing on their emerging potential as next-generation platforms for rapid, non-invasive and point-of-care diagnostics.

## 1. Introduction

The early and accurate detection of disease is of paramount importance and can only be possible with new developments in detection systems, allowing for better treatment outcomes and preventing death due to many life-threatening diseases (cancer, neurodegenerative disease, infectious diseases). Graphene transistor biosensors exhibit high sensitivity and selectivity with the label-free detection of cancer biomarkers, thereby opening the door to early diagnosis and personalized treatment—key elements in the progress towards real-time non-invasive diagnostics for cancer [1]. Their selective and stable characteristics have been improved by surface functionalization (SF) and electrical control, thus giving them diversified applications in the fields of healthcare, environment monitoring and point-of-care (POC) diagnostics based on two-dimensional (2D) nanomaterials with outstanding electronic properties [2,3]. Graphene, a two-dimensional carbon-based nanomaterial, presents remarkable electrical conductivities, a large surface area, mechanical strength and biocompatibility, which render it as an excellent material for biosensing applications [4,5].

Graphene field-effect transistor (GFET) biosensors work by measuring modulation in the electrical conduction of a graphene channel due to the interaction of target biomolecules (e.g., proteins, nucleic acids) with functionalized receptors immobilized on top of the graphene. The general device structure contains source, drain and gate contacts, while the graphene channel is placed in an electrolyte, enabling the label-free characterization of biochemicals in real time [6,7].

An important benefit of GFET-based detection is the ability to detect biomarkers from readily available biological fluids, such as blood, saliva and urine, which allows for diagnosis in a non-invasive manner [8]. This represents a major advance over traditional methods, such as colonoscopy and endoscopy, which can require sedation and tissue biopsies. Biomarkers are biological signatures of disease, which are particularly useful for diagnosing diseases in the early or pre-symptomatic stages [9,10].

The progress of GFET biosensors has expanded their applications for diagnosing various diseases. For example, GFET aptasensors not only are well suited for common medical diagnoses, but they also have sufficiently great potential in disease biomarker detection, as was illustrated via an OTA-specific sensor exhibiting a sensitivity of 1.4 pM within 10 s and exceptional sensitivity/real-time performance in high-speed mycotoxin sensing [11]. An avidin-modified GFET with the aid of avidin–biotin binding provided ultrasensitive biotin detection down to 0.37 pM and showed excellent specificity, as well as a possible application in point-of-care biomacromolecule diagnostics [12]. A linker-free patterned graphene FET demonstrated 2–4-fold enhanced sensitivity for tau protein detection (10 fg/mL–1 ng/mL) by directly immobilizing anti-tau antibodies on graphene edges, enabling efficient Alzheimer’s biomarker sensing in plasma [13]. A graphene oxide/graphene SGFET biosensor enabled the ultrasensitive, label-free detection of Alzheimer’s biomarker p-tau217 (10 fg/mL–100 pg/mL) with 18.6 mV/decade sensitivity and 0.991 linearity [14]. In the context of infectious diseases, GFETs have been employed to detect HIV-1 RNA and even SARS-CoV-2 spike proteins [15,16].

Innovations in fabrication, such as doping, have significantly enhanced GFET biosensor performance. A gold nanoparticle-decorated graphene field-effect transistor (GFET) functionalized with peptide nucleic acid probes enabled label-free miRNA detection with 10 fM sensitivity, precise single-base discrimination, and reliable performance in serum, demonstrating strong potential for early cancer and gene-related disease diagnostics [17]. An ultrasensitive poly-L-lysine-functionalized graphene field-effect transistor (PGFET) biosensor was developed for rapid breast cancer miRNA and SARS-CoV-2 RNA detection, achieving 1 fM sensitivity, a five-order detection range, and 113% improved performance within 20 min using minimal serum or swab samples [18]. A gold nanoparticle-decorated GFET nanosensor functionalized with phosphorodiamidate morpholino oligo (PMO) probes enabled rapid, unamplified SARS-CoV-2 RNA detection with detection limits of 0.37 fM (PBS), 2.29 fM (throat swab), and 3.99 fM (serum), providing 2 min responses and 0.92 Kappa agreement for precise COVID-19 diagnosis [19]. A platinum nanoparticle-decorated reduced graphene oxide FET biosensor integrated with a microfilter enabled label-free, highly sensitive BNP detection in whole blood, achieving a 100 fM detection limit for early heart failure diagnosis [20]. These advances highlight the utility of GFETs and their potential to serve as a platform technology for point-of-care early diagnosis with high sensitivity.

This review presents an overview of functionalization, detection mechanisms and technological applications relevant to GFET biosensors. Table 1 lists a few examples of GFET-based configurations with the corresponding biomarkers and detection limits reported for the various setups from the literature [21,22,23,24,25,26,27,28]. Conventional biosensors like ELISA achieve detection limits of 1–100 nM for disease biomarkers, while SERS/colorimetric methods reach 3–5 ng/mL (~20–33 pM) for clusterin; in contrast, GFETs demonstrate fM–aM sensitivity (e.g., 4 fM for clusterin, 2.62 fM for ER-α), representing superior performance [6,7,21,23].

**Figure 1:**
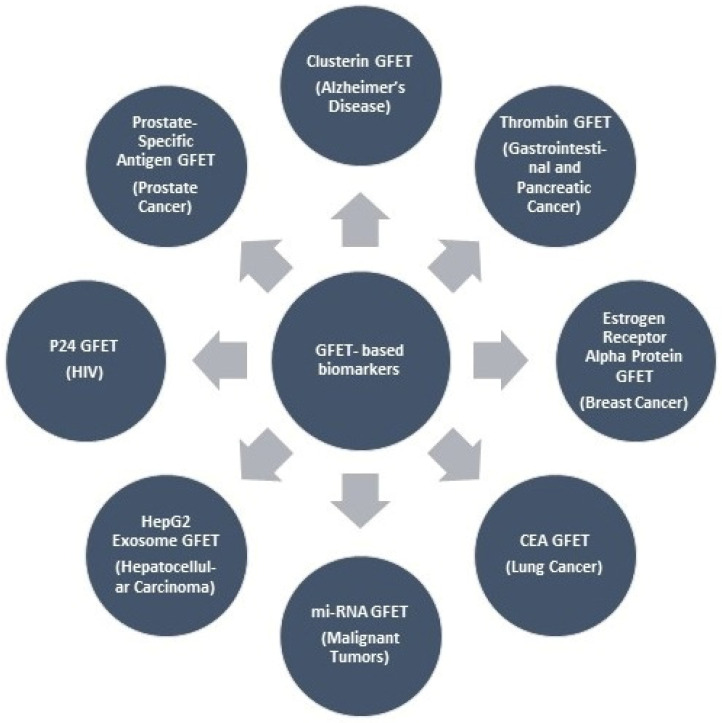
Classification of disease-specific GFET biosensors according to their target disease biomarkers.

The clinical importance and scope of GFET biosensing regarding diagnosis are measured using the comprehensive approach illustrated in Figure 1, which outlines the clinical value chain that maps disease biomarkers to their respective health states. The versatility of GFET biosensors along this broad disease spectrum is a testament to their innate flexibility and the tunability of surface functionalization methodologies. The modular nature of GFET fabrication and functionalization allows such sensors to be designed for specific sensing targets, positioning them as suitable candidates for integration into multiplexed diagnostic platforms that can detect multiple biomarkers at a time from the same sample.

This review emphasized the comprehensive content of many diseases such as gastrointestinal disease, pancreatic cancer, malignant tumors, Alzheimer’s disease, breast cancer, lung cancer, prostate cancer, hepatocellular carcinoma, and chronic obstructive pulmonary disease. As a whole, this review provides a critical assessment of GFET biosensor devices for disease biomarkers that have potential for translation to market and the clinical utility of diagnostics as healthcare tools.

## 2. Working Principles of GFETs and Their Interaction with Biosensitivity

**Figure 2:**
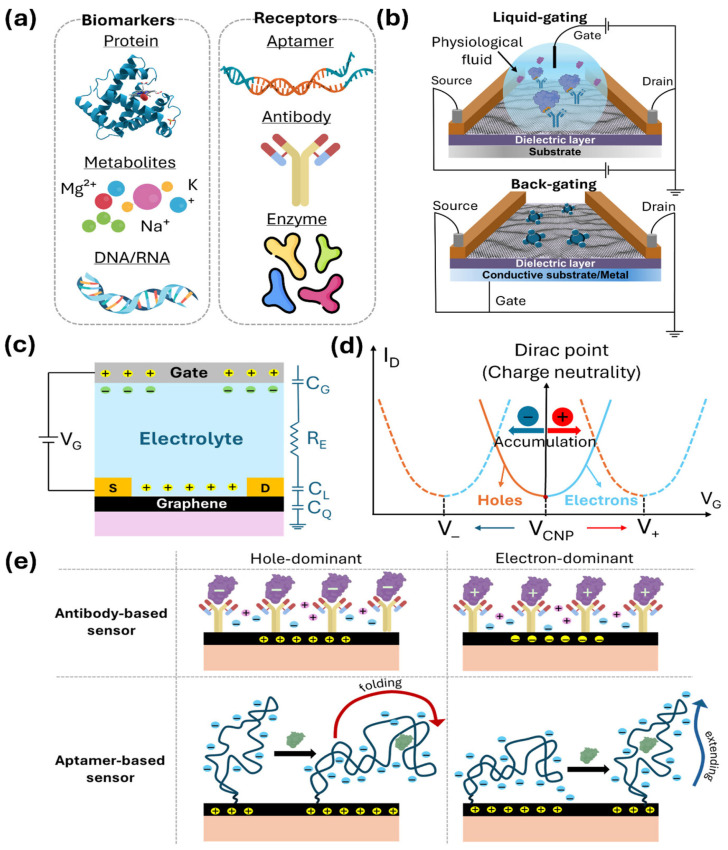
Fundamental operating principles and sensing mechanism of GFET biosensors: (**a**) Common analytes and probes used in GFETs. (**b**) Configuration of liquid-gated GFET (**top**) commonly used for electrolyte-based biosensing and back-gate GFET (**bottom**) commonly used for gas sensing. (**c**) Graphene–electrolyte–gate interface capacitance caused by charged analytes. (**d**) Dirac point shift in graphene channel toward different charged analytes. (**e**) Binding event via aptamer and antibody causing electrostatic induction in graphene channel [29].

GFET biosensor performance is strongly governed by the structural form of graphene, including layer number, defect density, and synthesis method. Single-layer graphene (SLG) provides high carrier mobility and strong electrostatic coupling, enabling ultrasensitive, label-free detection, but it is more susceptible to environmental noise and signal drift. Few-layer graphene (FLG) offers reduced sensitivity due to partial charge screening, yet it improves mechanical stability, noise characteristics, and device reproducibility, making it suitable for practical sensing conditions. Reduced graphene oxide (rGO), while exhibiting lower mobility due to structural defects, facilitates efficient biomolecule immobilization because of its abundant functional groups and is therefore favored in cost-effective and scalable sensing platforms [4,5,6,7]. The synthesis route further modulates these properties: CVD graphene enables large-area integration but introduces grain boundaries, exfoliated graphene provides superior intrinsic quality with limited scalability, and solution-processed derivatives prioritize manufacturability over electronic performance. Graphene possesses high carrier mobility, a large surface-to-volume ratio, and a tunable Fermi level, making it highly suitable for field-effect transistor-based biosensing. These properties enable the sensitive, label-free detection of biomolecular interactions [5,6,7]. Graphene field-effect transistors (GFETs) are the modified version of FETs which use graphene as a working channel instead of silicon for functionalization. FETs work on the principle, where a voltage-controlled device regulates the flow of current through a semiconductor channel using an electric field applied at the gate terminal, as shown in Figure 2 [29]. The biomarkers are the same, like the proteins and metabolites shown in Figure 2a, and the GFET comprises three terminals, the source, drain, and gate, with a graphene channel bridging the source and drain, as shown in Figure 2b,c. The current is the lowest at the Dirac point, where both carriers are equal (Figure 2d), and the gate voltage changes the Fermi level of graphene (Figure 2e), switching between electron and hole dominance, fostering efficient sensor activity.

Graphene, being a two-dimensional monolayer of carbon atoms arranged in a hexagonal lattice, exhibits exceptional electronic properties, including very high electron mobility, superior electrical conductivity, and significant carrier velocity at room temperature [6,29]. In a GFET, this graphene channel possesses defined length and width dimensions; when a voltage is applied between the drain and source terminals, current flows through the graphene channel. The gate terminal is electrically isolated from the graphene channel and is utilized to apply a voltage which induces an electric field to modify the carrier concentration in the graphene channel, thereby modulating current flow through; this modulation mechanism forms the fundamental operating principle of GFETs.

Graphene field-effect transistors (GFETs) work by varying the charge carrier density in a graphene channel with an external gate voltage, and thus they can sense surface interactions, such as biomolecule adsorption [8]. The fundamental mechanism is the electric field effect; gate voltage changes the Fermi level of graphene and thus modulates the carrier concentration (and therefore current) flowing in between the source and drain.

### 2.1. Charge Carrier Modulation in GFETs

In GFET biosensors, biomolecular interactions are transduced into electrical signals through the modulation of charge carrier density in the graphene channel induced by electrostatic gating effects. The source–drain current (*I_DS_*) in a GFET is directly dependent on the gate-induced carrier density *n*, mobility *μ*, and channel geometry. In the linear regime, the current can be approximated as follows:

(1) $$I_{DS}=\mu C_{g}\frac{W}{L}(V_{G}-V_{Dirac})V_{DS}$$

where *μ* = charge carrier mobility (cm^2^/V·s); *C_g_* = gate capacitance per unit area (F/cm^2^); *W* and *L* = width and length of the graphene channel; *V_G_* = gate voltage; *V_DS_* = drain–source voltage; *V_Dirac_* = gate voltage at the Dirac point (minimum conductivity point).

The position of the Dirac point, *V_Dirac_*, shifts in response to charge transfer events or electrostatic gating effects caused by biomolecular adsorption on the graphene surface [17,25,26].

### 2.2. Gate Capacitance Considerations

When operated in liquid-gated configurations, GFET biosensors rely on the formation of an electrical double layer at the graphene–electrolyte interface, resulting in an exceptionally high gate capacitance. The nanometer-scale thickness of the EDL leads to an exceptionally high gate capacitance, which allows for the efficient modulation of graphene carrier density at low gate voltages. This mechanism underpins the high sensitivity of GFETs, as biomolecular adsorption within or near the EDL directly perturbs the local electrostatic environment and shifts the device transfer characteristics.

In liquid-gated GFETs used for biosensing, gate capacitance (*C_g_*) is largely dominated by the electrical double layer (EDL) at the graphene electrolyte interface:

(2) $$C_{g}=\frac{\varepsilon_{r}\varepsilon_{0}}{\lambda_{D}}$$

where *ε_r_* = relative permittivity of the electrolyte; *ε*_0_ = vacuum permittivity; *λ_D_* = Debye length, typically a few nanometers in biological fluids.

The high capacitance of the EDL enables the modulation of carrier density with small changes in gate voltage, contributing to the high sensitivity of GFETs [17,25,26,27].

### 2.3. Transconductance as a Sensitivity Metric

Transconductance (*g_m_*), defined as the change in *I_DS_* per unit change in gate voltage, is a key figure of merit for GFET biosensors:

(3) $$g_{m}=\frac{{dI}_{DS}}{{dV}_{G}}=\mu C_{g}\frac{W}{L}V_{DS}$$

A larger value of gm indicates more sensitivity to variations in surface charge and is essential for the ultrasensitive detection of low-abundance analytes [17,25,26,27,28,29].

### 2.4. Biomolecular Interaction and Doping Effects

From a biosensing point of view, the analytical performance of GFETs does not only result from graphene’s high carrier mobility but is also due to the strong electrostatic interaction between surface charge perturbations and channel conductance. In stark contrast to classical electrochemical sensors based on faradaic reactions, GFET devices directly transduce biomolecular binding signatures into a modulation in conductance by means of FET gating [30]. This provides the benefit of label-free, real-time signal capture and offers operation at very-low-concentration analytes with minimal charge screening. Nonetheless, this same electrostatic transduction scheme also introduces restrictions at the most basic level. Debye screening also limits the effective sensing distance to a couple of nanometers in physiological electrolytes, limiting sensitivity toward larger biomolecules and nonoptimally oriented receptors. Moreover, GFETs that consist of practical probe design, surface chemistry, and electrolyte status show vastly different performance. As a result, even though GFETs have excellent intrinsic sensitivity, the analytical benefit of them is now regarded as being realized in some specific conditions rather than generally, and specialized design is required for their target operation [6,30].

An externally applied electrostatic field can be felt locally when a charged biomolecule (protein, DNA, aptamer–target complex) is adsorbed on the functionalized graphene surface and then causes the modification of the carrier distribution. The effect of this interaction can be described as a shift in the Dirac voltage:

(4) $${\Delta V}_{Dirac}=\frac{qN_{s}}{C_{g}}$$

where *q* = elementary charge; *N_s_* = surface density of adsorbed biomolecules.

The direction and magnitude of the Dirac point shift depend on the net charge of the analyte at physiological pH.

### 2.5. Label-Free and Real-Time Detection Advantages

In contrast to traditional biosensors that utilize fluorescent or enzymatic labels, GFET biosensors are label-free. The direct transduction of binding events into electrical signals eliminates additional steps and enables the real-time monitoring of biomolecular interactions with sub-picomolar sensitivity. Additionally, the ambipolar nature of graphene allows for dual-mode sensing for both electron-donating and electron-accepting targets.

The sensing mechanism in GFETs originates from charge transfer and electrostatic gating effects induced by target molecules binding to the graphene surface or to receptor molecules functionalized on the channel. The drain current $I_{D}$ of a GFET can be expressed as defined in Equation (1).

The binding of charged biomolecules modifies the local electrostatic potential, leading to a measurable shift in the Dirac voltage. A key advantage of graphene is its ambipolar conduction, allowing for both electron (n-type) and hole (p-type) transport. As a result, GFETs support dual-mode sensing, where electron-donating species shift the Dirac point toward negative gate voltages, while electron-withdrawing species induce positive shifts. This ambipolar response broadens the range of detectable analytes and enhances discrimination capability without modifying device architecture. Furthermore, real-time sensing is enabled by the continuous monitoring of $I_{D}(t)$, allowing for the extraction of binding kinetics using Langmuir adsorption models:

(5) $$\frac{d\theta }{dt}=k_{on}C(1-\theta )-k_{off}\theta$$

where θ is the fractional surface coverage, C is the analyte concentration, and $k_{on}$ and $k_{off}$ are the association and dissociation rate constants. This capability positions GFETs as powerful platforms for dynamic biomolecular interaction analysis beyond end-point detection.

### 2.6. Nucleic Acid Detection

The GFET-based detection of nucleic acids is of significance importance in the field of forensic environmental monitoring and customized medications. It excels in label-free nucleic acid sensing by evaluating shifts in their intrinsic charges. One study showed the use of probe DNA or PNA immobilized on graphene channels, and such a GFET enabled ultrahigh-sensitivity detection in buffer and human serum samples down to millimeter-scale structures at 600 zM and 20 aM, respectively, which are equivalent to 18 and 600 nucleic acid molecules [31].

Typically, single-stranded DNA (ssDNA) or peptide nucleic acid (PNA) probes are functionalized onto graphene via π–π stacking or linker chemistries. Upon target hybridization, the effective surface charge density changes, producing a detectable Dirac point shift:

(6) $$\Delta V_{Dirac}\propto \frac{qN_{DNA}}{C_{EDL}}$$

where $N_{DNA}$ is the number of hybridized nucleic acid molecules, and $C_{EDL}$ is the electric double-layer capacitance at the graphene–electrolyte interface.

The sensitivity of nucleic acid detection is strongly influenced by Debye screening, characterized by the Debye length $\lambda_{D}$:

(7) $$\lambda_{D}=\sqrt{\frac{\varepsilon k_{B}T}{2N_{A}e^{2}I}}$$

where ε is dielectric permittivity, $k_{B}$ is Boltzmann’s constant, T is temperature, $N_{A}$ is Avogadro’s number, e is electron charge, and I is ionic strength. To overcome screening in physiological media, ultra-short probes (e.g., PNA) and optimized buffer conditions are employed to keep the binding charges within $\lambda_{D}$, preserving signal integrity.

Remarkably, recent studies have demonstrated GFET-based nucleic acid detection down to 600 zM in buffer and 20 aM in human serum, corresponding to approximately 18 and 600 target molecules, respectively. The limit of detection (LOD) is commonly defined as follows:

(8) $$\mathrm{LOD}=\frac{3\sigma }{S}$$

where σ is the standard deviation of baseline noise, and S is sensor sensitivity (Dirac shift per concentration unit). The atomically thin nature of graphene ensures that nearly all charge perturbations occur within the conduction channel, enabling detection at the single- to few-molecule level [6].

These attributes establish GFETs as a highly promising platform for ultralow-concentration nucleic acid diagnostics, offering label-free operation, real-time readout, and exceptional sensitivity even in complex biological matrices.

### 2.7. Protein–GFET Interactions and Surface Functionalization

Protein detection in graphene field-effect transistors (GFETs) relies on field-effect modulation arising from electrostatic and charge transfer interactions at the graphene–electrolyte interface. Upon protein binding, the local charge distribution and dipole moment of the biomolecule perturb the interfacial electric field, leading to shifts in carrier density and the Dirac point of the graphene channel. In liquid-gated GFETs, this mechanism enables the label-free transduction of binding events; however, effective signal generation critically depends on the distance between protein charge centers and the graphene surface due to Debye screening in physiological media [32].

Because pristine graphene is chemically inert and prone to nonspecific adsorption, surface functionalization is essential for selective protein recognition and stable signal transduction [33]. Noncovalent strategies, such as π–π stacking using aromatic linkers, preserve graphene’s sp^2^ lattice and high carrier mobility while enabling receptor immobilization. Covalent functionalization offers greater interfacial stability but introduces lattice defects that can degrade electronic performance [34]. Protein orientation and linker length strongly influence sensitivity, as misalignment or excessive spacing weakens electrostatic coupling. To suppress background signals in complex biofluids, passivation layers (e.g., PEG or BSA) are commonly employed [35]. Overall, optimized interface engineering that balances electronic integrity, biointerface stability, and molecular accessibility is key to achieving sensitive and reproducible protein sensing in GFET platforms. Protein binding induces a shift in the Dirac voltage proportional to the effective surface charge density, as expressed in Equation (6), representing the net charge contribution and dipole-induced electrostatic effect of bound protein molecules. Importantly, proteins often carry heterogeneous charge distributions, meaning that both net charge and orientation-dependent dipole moments contribute to the signals observed by biosensors [36,37].

In physiological electrolytes, effective protein sensing is constrained by Debye screening, which attenuates electrostatic interactions beyond the Debye length $\lambda_{D}$ as expressed in Equation (7). At typical ionic strengths (∼150 mM), $\lambda_{D}$ is approximately 0.7–1 nm, significantly shorter than the dimensions of most proteins. The effective electrostatic potential $\psi \left(d\right)$ sensed by graphene decays exponentially with the distance d between the protein charge center and the graphene surface:

(9) $$\psi (d)=\psi_{0}\;\exp\left(-\frac{d}{\lambda_{D}}\right)$$

Consequently, linker length, receptor architecture, and protein orientation directly determine sensing efficiency. Excessively long linkers or unfavorable orientation can drastically reduce the transduced signal, even for biosensors based on high-affinity binding events [6,29,30,31,32,33,34,35,36,37].

Pristine graphene is chemically inert and susceptible to nonspecific adsorption, necessitating surface functionalization to achieve selective and stable protein sensing. Functionalization strategies fall broadly into noncovalent and covalent approaches [31,32,33,34,35,36,37].

Noncovalent functionalization typically employs aromatic linker molecules (e.g., pyrene derivatives) that attach to graphene via π–π stacking. This approach preserves the sp^2^ carbon lattice, maintaining high carrier mobility and low noise. The surface charge density contributed by immobilized receptors can be described as follows:

(10) $$\sigma_{eff}=qN_{r}\theta_{r}$$

where $N_{r}$ is receptor density, and $\theta_{r}$ is fractional surface coverage.

Covalent functionalization, often involving diazonium or plasma-based chemistries, provides enhanced chemical robustness but introduces lattice defects that increase scattering and reduce mobility:

(11) $$\mu \propto \frac{1}{N_{defects}}$$

As a result, covalent approaches may compromise device performance if defect density is not carefully controlled.

The orientation of immobilized proteins is a critical determinant of sensitivity. Random adsorption can place charged domains beyond the Debye length, whereas oriented immobilization (e.g., via protein A/G or engineered affinity tags) positions active binding sites closer to the graphene surface, maximizing electrostatic coupling [32,33,34,35,36,37].

In complex biological matrices, nonspecific adsorption introduces background noise and signal drift. Passivation layers such as polyethylene glycol (PEG) or bovine serum albumin (BSA) are employed to suppress nonspecific interactions. The effectiveness of antifouling layers can be quantified by the signal-to-noise ratio (SNR):

(12) $$\mathrm{SNR}=\frac{\Delta I_{signal}}{\sigma_{baseline}}$$

where $\sigma_{baseline}$ represents current fluctuations due to nonspecific adsorption and ionic noise.

Overall, optimized interface engineering—balancing graphene electronic integrity, stable biofunctionalization, minimized Debye screening, and antifouling performance—is essential for achieving sensitive, selective, and reproducible protein detection in GFET-based biosensors [36,37].

## 3. Discussion of GFET-Related Disease Biomarkers

The GFET configuration’s modular approach enables the detection of a wide array of biomarkers across various disease categories, including neurodegenerative, oncological, pulmonary, viral, and endocrine disorders. The use of noncovalent immobilization strategies and optimized gating configurations contributes to the ultralow detection limits and rapid signal response characteristic of GFET biosensors.

### 3.1. Clusterin Biomarker

Diseases like Alzheimer’s are of a progressive nature, and they lead to memory loss with time due to the accumulation of toxic species of proteins in the brain [38]. It affects the central part of the nervous system, leading to several structural changes in the brain, making it neuron-deficient and leading to the build-up of proteins like amyloid beta, thereby hindering the day-to-day activities of affected humans. Such diseases need efficient detection for timely treatment, which can be done by the detection of clusterin as this protein is present in the brain and body fluids, and it prevents the aggregation of amyloid beta [39]. There are few studies on the detection methods of the clusterin biomarker, and one study presents the use of a surface-enhanced Raman scattering (SERS) immunoassay built on an aluminum foil substrate for the sensitive detection of the clusterin biomarker. The aluminum foil, especially its matte side, demonstrated superior performance compared to other substrates like gold and silicon. The detection limit was as low as 3.0 ng/mL, showing a reliable quantitative response over a concentration range from 1 to 1000 ng/mL [40]. The colorimetric aptasensor for clusterin detection in urine using gold nanoparticles yielded a limit of detection of 5.37 pg/mL, with a rapid (15 min) visible color change from red to gray, correlating with biomarker concentration. Aptamers provided high selectivity, and this method was demonstrated as being effective in clinical urine samples, offering a simple, label-free, and cost-effective alternative to antibody-based sensors. This method emphasized ease of use and clinical applicability but did not reach the ultrasensitive levels of GFETs [41]. GFET biosensors provide the highest sensitivity and electrical label-free detection ideally suited for early-stage diagnostics and multiplexing, as studies of GFET biosensors have shown, where a monolayer of graphene was fabricated via chemical vapor deposition on a Si/SiO_2_ sheet-like substrate with a thickness of 300 nm, which was effectively developed for the sensitive detection of the clusterin biomarker and verified by electrical and spectroscopic studies. Clusterin is a key protein implicated in Alzheimer’s disease. The fabrication of a GFET on Si/SiO_2_ substrates involves two main aspects, consisting of the creation of graphene channels via functionalization and the making of source, drain, and voltage electrodes. Anti-clusterin antibodies are also fabricated on the substrate, and these sensors utilize electrical resistance changes to achieve a detection limit as low as 300 fg/mL (4 femtomoles), covering a concentration range from 1 to 100 pg/mL. The sensors exhibit a 30% decrease in resistance from bare to annealed conditions, and upon clusterin binding, there was an increase in resistance ∼118%, demonstrating high sensitivity and specificity, with minimal response to unrelated biomolecules. The sensors’ electrical characteristics, including low contact resistance and high carrier mobility, contribute to theit reproducibility and reliability. These attributes make GFET biosensors a promising platform for the rapid, low-cost, and precise detection of biomarkers relevant to neurodegenerative and other diseases, with potential applications extending beyond Alzheimer’s diagnosis [21].

### 3.2. Thrombin Biomarker

Biomarkers have growing importance due to their portability and modern lifestyle demands, including constant monitoring. Thrombin is a critical protease enzyme found in blood plasma that plays an essential role in the coagulation cascade, making it a valuable biomarker for blood-related disorders. It catalyzes the conversion of fibrinogen to fibrin, leading to blood clot formation, which is vital for wound healing and maintaining hemostasis. Aberrant thrombin levels can indicate pathological states such as thrombosis, cardiovascular diseases, and stroke, underscoring its clinical significance. Monitoring thrombin concentrations provides key insights for diagnosing and managing these conditions [42].

Thrombin has rarely been reported to be detected by via GFETs, as per the literature. Only one study utilizing GFET biosensors has been reported for the detection of thrombin, demonstrating significant potential for ultrasensitive and rapid thrombin detection. Khan et al. developed an integrated microfluidic GFET employing the lab-on-a-chip concept, which used the chemical vapor deposition (CVD) technique for fabrication, and they achieved a detection limit of 2.6 picomoles. CVD fabrication offers high repeatability for industrial-scale production along with enabling selective and real-time monitoring [22].

### 3.3. Estrogen Receptor α (ERα) Protein Biomarker

**Figure 3:**
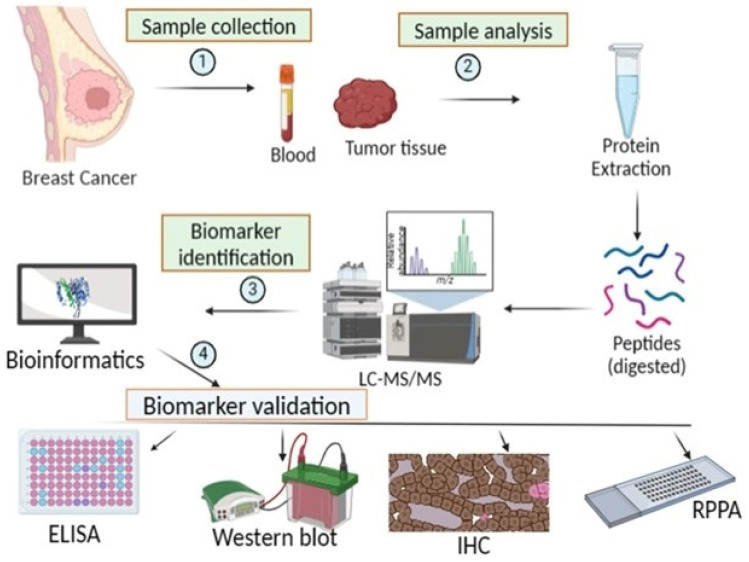
The biomarker detection and validation process of protein biomarkers for breast cancer [45].

Estrogens are crucial for proper reproductive functions for both males and females, and particularly, estrogen receptor alpha (ERα) promotes the proliferation of breast epithelial cells via the activation of estrogen receptors [43]. This increased cell proliferation raises the likelihood of mutations during DNA replication, which can accumulate and lead to malignant treatment for breast cancer, requiring diagnosis in its early stages, which is possible if there is a convenient and cost-effective detection system [44]. So far, mammography has been used for its diagnosis, but it has its limitations. A recent approach to the detection of protein biomarkers for breast cancer is shown in Figure 3, consisting of how the samples of blood and tissue from the breast are taken and processed for detection and validation [45].

A liquid-gated GFET-based biosensor was reported for the detection of estrogen receptor α (ERα). The fabricated synthetic drug molecule-modified graphene surfaces to selectively capture ERα proteins. The sensor showed ultrahigh sensitivity with the capacity to perform at a lower detection limit of 2.62 fM, along with the ability to distinguish the targeted protein even in complex environments [23].

### 3.4. Carcinoembryonic Antigen (CEA) Biomarker

Cancer is a leading cause of death worldwide, characterized by multiple types, many of which can be fatal if diagnosed at late stages. Early detection is critical, as treatment options become more effective and less complex when cancer is identified early. Biomarkers, such as Carcinoembryonic antigen (CEA), play a vital role in cancer detection and monitoring. Traditional immunoassay methods have been widely used for biomarker detection, but recent advances in biosensor technologies, including graphene field-effect transistor (GFET) biosensors, offer enhanced sensitivity, specificity, and rapid analysis. GFET-based sensors for CEA provide a low-cost, label-free platform capable of detecting ultralow concentrations of this biomarker, enabling earlier and more precise diagnosis and ongoing monitoring of cancer progression [46]. A GFET biomarker for the detection of CEA was fabricated by the functionalization of graphene via a one-step method, in which a nano-denatured bovine serum albumin (nano-dBSA) solution was heated so that it could cast a firm layer over graphene, and such functionalized graphene displayed significant sensitivity towards CEA. Anti-CEA was also developed. When a sample containing CEA was introduced, the antigen was bound to the anti-CEA antibody immobilized on the graphene channel. This specific recognition event caused a measurable change in the device’s electrical properties, detected as a change in drain–source current. The sensor thus converted the biochemical interaction received from the receptor binding CEA and anti-CEA in the electrolyte into a quantifiable electrical signal, enabling the highly sensitive (down to 337.58 fg/mL) and specific detection of CEA for cancer diagnosis and monitoring applications [24].

### 3.5. MicroRNA (miRNA) Biomarker

Acute myocardial infarction (AMI) is among the most fatal cardiovascular disorders, leading to irreversible myocardial damage and potential heart failure [47,48]. Early and immediate diagnosis is essential to reduce mortality, increase survival, and improve recovery. Cardiac troponin T (cTnT) is known for its role as a biomarker of AMI; nevertheless, it has low specificity due to the fact that cTnT values can be increased in other cardiac states and conditions of the heart such as coronary artery disease [49]. In addition, protein biomarkers experience a slow rise and have a long half-life, limiting their utility as early diagnostic markers of AMI. Accordingly, more and more focus has been placed on upstream molecular biomarkers such as microRNAs (miRNAs), which have advantages in earlier detection in terms of myocardial injury with superior sensitivity or lag time improvement and so forth.

Traditional methods of miRNA detection (such as RNA imprint, RT-qPCR and microarray assays) are limited by low sensitivity, long detection time, high cost and complicated operation [50,51]. RNA imprinting is a method that requires high sample volume, and although RT-qPCR is specific, it involves long hands-on time. The requirements of probe labeling, bulky instrumentation and personnel skill make microarray analysis tools less portable [52,53]. Recent semiconductor miniaturization is leading in the area of field-effect transistor (FET) sensors based on two-dimensional nanomaterials in terms of being the most promising technology in integrated, low-cost and ultrasensitive miRNA detection. A three-dimensional graphene structure grown on a nickel foam by chemical vapor deposition was used as a conductive channel for miRNA detection using field-effect transistor biosensors, with a limit of detection of 100 pM and linear range of 100 pM–100 nM [54].

MicroRNA-208a (miR-208a) is a cardiac-specific biomarker that can be used to diagnose acute myocardial infarction (AMI) earlier than traditional protein markers can. A liquid-gated GFET biosensor was fabricated for fast and ultrasensitive detection. Integrated with a portable electronic readout system, the sensor presented a detection range of 0.01–1 pM and a remarkable LOD of 5.3 fM within a 40 min response time. This device exhibited excellent real-time sensitivity for miR-208a with a detection limit of 1.7 pM and provided a general method for developing GFET-based biosensors for the detection of other disease biomarkers, which significantly enriches early diagnosis and personalized healthcare applications [55].

A peptide nucleic acid (PNA)-functionalized laser-induced graphene biosensor was developed using a single-step fabrication and probe functionalization strategy with smartphone-based readout [56]. The device detected prostate cancer biomarker hsa-miR-141 with 0.6 aM sensitivity. This amplification-free, portable, and enzyme-independent platform demonstrates strong potential for low-cost point-of-care clinical diagnostics.

**Figure 4:**
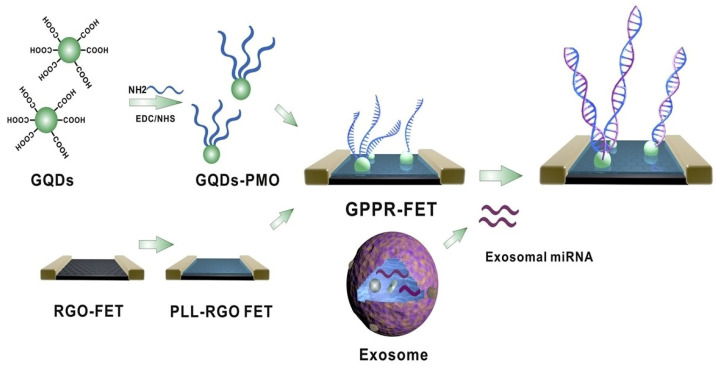
Schematic representation of PMO–GQD hybrid immobilized on polylysine-coated rGO-FET that detects exosomal miRNAs through probe hybridization [57].

An ultrasensitive reduced graphene oxide (rGO)-based field-effect transistor (rGO-FET) biosensor functionalized with phosphorodiamidate morpholino oligomer–graphene quantum dot (PMO–GQD) hybrids was developed for exosomal miRNA detection [57]. A schematic illustration of the PMO–GQD-functionalized rGO-FET biosensor is displayed in Figure 4, showing polylysine-assisted immobilization, exosomal miRNA hybridization on the chip surface, and the resulting Dirac point shift due to charge-induced carrier modulation. The device, with a polylysine interlayer for stable binding, achieved an 85 aM detection limit and high specificity, successfully distinguishing breast cancer plasma samples from healthy controls, demonstrating strong potential for early breast cancer diagnosis.

A flexible, ultrasensitive field-effect transistor (FET) biosensor featuring defect-free van der Waals contacts was developed for rapid miRNA-155 detection [58]. The device achieved an ultralow detection limit of 1.92 fM, a broad linear range of 10 fM–100 pM, and a 10 min response time that is five times more sensitive than conventional graphene FETs. It maintained performance after 100 bending cycles and successfully distinguished miRNA levels in patient serum and sweat, highlighting its potential for practical, non-invasive, and wearable cancer diagnostics and continuous health monitoring applications.

### 3.6. HepG2 Exosome Biomarker

Graphene field-effect transistors (GFETs) have been successfully implemented for the sensitive detection of HepG2 exosomes using an aptamer-based biosensing platform. Chen et al. reported an innovative aptamer (LZH8-TDN) integrating artificial nucleotides into a tetrahedral DNA nanostructure (TDN) for rapid hepatocellular carcinoma (HCC) diagnosis using a GFET within 9 min [25]. The devices were operated using a liquid gate configuration, utilizing a Ag/AgCl reference electrode to control the gate voltage [59,60]. This TDN was self-assembled from four amino-terminated single-stranded DNA oligonucleotides and served as a three-dimensional scaffold for aptamer presentation. The TDNs were anchored to graphene using 1-pyrenebutyric acid N-hydroxysuccinimide ester (PASE), which forms covalent bonds with the amino groups of the DNA strands and π–π stacked noncovalent interactions with the graphene lattice [61,62]. Exosomes, extracted from HepG2 liver cancer cells through ultracentrifugation, were employed as the detection targets. The graphene channel, synthesized by chemical vapor deposition and coupled with Au electrodes, was functionalized via PASE linkers enabling π–π interactions with LZH8-TDN. This configuration enhanced aptamer stability, sensitivity, and nanoscale selectivity. Notably, the aptasensor exhibited a five-fold higher signal in HCC samples compared to healthy controls, highlighting strong clinical potential [25].

There are several types of nanomaterials examined for hepatocellular carcinoma (HCC); among them, graphene oxide (GO) nanosheets have received particular attention as a promising anti-cancer material. In contrast to metallic nanoparticles, which frequently induce apoptosis by ROS production or in a caspase-dependent manner, GO-induced cytotoxicity in HepG2 cells occurred as an arrest in the S phase of the cell cycle with DNA fragmentation and the upregulation of Bax but without a change in the expression level of p53 or caspase-3 [63]. This is consistent with a caspase-independent but endogenous apoptotic pathway. This is one of the most unique features that distinguishes GO from other nanoplatforms and indicates the potential of GO as a new therapeutic modality for HCC treatment.

**Figure 5:**
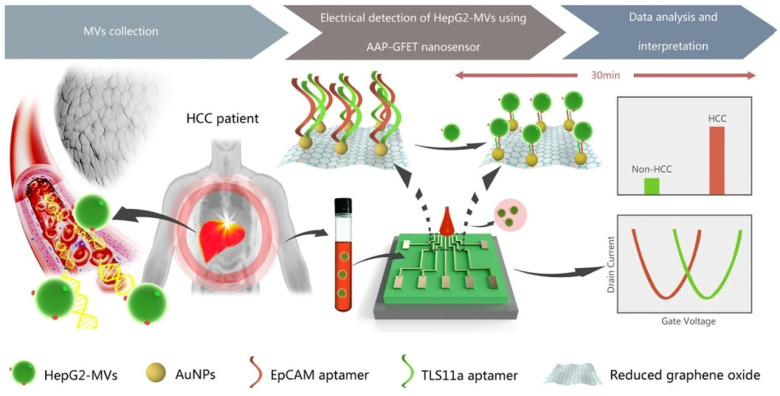
Schematic illustration for electrical detection of hepatocellular carcinoma (HCC)-derived microvesicles (MVs) using AAP-GFET nanosensor [64].

Cancer-derived microvesicles (MVs), nanoscale vesicles released into the extracellular space, are increasingly recognized as valuable biomarkers for early cancer detection, yet their reliable identification remains challenging. In another study, a dual-aptamer-modified reduced graphene oxide (rGO) field-effect transistor (AAP-GFET), which was fabricated using gold nanoparticles (AuNPs) for the highly sensitive and label-free monitoring of HepG2-derived MVs, was utilized (as depicted in Figure 5) [64]. This bifuxial aptamer interface was characterized by high sensitivity, a wide dynamic range and remarkable specificity in the presence of irrelevant proteins, as well as MVs from non-hepatic cells. Clinical practice showed their good ability to discriminate between HCC patients and healthy controls, emphasizing their diagnostic potential.

The biotinylation of microvesicles (MVs) was validated using flow cytometry and fluorescence colocalization. HepG2 cells, the parental source of biotinylated MVs (B-MVs), exhibited nearly complete surface modification, with 99.5% biotinylation efficiency after incubation with streptavidin-Cy3 (SA-Cy3), consistent with earlier reports. MVs secreted from these cells also showed high labeling efficiency (92.6%), confirming effective biotin incorporation. Fluorescence studies also confirmed the colocalization of SA-Cy3-labeled B-MVs with DiO-stained vesicles; however, MVs from the control did not overlap, indicating a lack of biotin. These findings verify the successful biotinylation of MVs, which securely supports the use of these MVs for biosensor applications based on biotin–streptavidin affinity technology [65].

HCC, a leading form of liver cancer, necessitates early and accurate detection for effective treatment outcomes [66,67,68]. A GFET-based biosensor that has an exon-inhibiting aptamer-modified VEGF and id anchored by a tetrahedral DNA nanostructure was developed by Chen et al. The modified nanostructure not only had high specificity for the target but also enabled the stable immobilization of those at the graphene surface [25].

HCC is still a challenging disease to control because of the limited satisfaction with current therapies. It has been shown that the exosomes derived from stem cells when combined with etoposide (ETO) can synergistically promote HepG2 cell apoptosis. Co-administration promoted the activation of caspase-3 and -9 and elevated the Bax/Bcl-2 ratio and the upregulation of p53 more than single treatments [69]. These results support the use of exosome–chemotherapy cocktails as a potential approach to enhancing HCC treatment efficiency.

Graphene-driven electrochemical biosensors have recently become potential candidates in the detection of HCC markers, which allows for simple, sensitive and specific measurements. More recently, they have been applied in the detection of Golgi protein-73, alpha-fetoprotein, exosomes and microRNA-122 [70]. Exosomes, initially thought of as cellular waste, have emerged as stable, nanosize vesicles containing proteins, lipids, and nucleic acids essential for intercellular communication. Their disease-specific cargo suggests these nanoparticles as being potential diagnostic and prognostic biomarkers in cancer and neurodegenerative, cardiovascular and autoimmune diseases, being applicable to liquid biopsies, immunity and regenerative medicine [71].

### 3.7. Interleukin-6 (IL-6) Biomarker

GFETs have also been used for the detection of interleukin-6 (IL-6), as an important cytokine in inflammatory and immune responses [72]. Hao et al. integrated a portable graphene-based GFET nanosensor for the highly sensitive detection of interleukin-6 (IL-6), which is a very important biomarker related to various diseases [26]. The chemical vapor deposistion (CVD) of a graphene-based device with a buried-gate structure was functionalized with PASE linkers for anchoring IL-6-specific aptamers. The aptamers were bound to the graphene channel and led to charge redistribution, inducing current changes. Functionalization was confirmed by Raman spectra indicating π–π interactions with PASE. This nanosensor achieved label-free IL-6 detection in saliva with a detection limit of 12 pM, highlighting its promise for early non-invasive diagnostics [26].

Aptameric GFETs (A-GFETs) typically rely on pyrene-based linkers such as PASE, which attach to graphene via π–π stacking; however, conventional immobilization produces random and limited densities, reducing sensitivity. A novel electric field-assisted approach enabled controlled linker orientation and density, thereby enhancing aptamer loading and sensor response. Using this strategy, A-GFETs achieved ultralow detection limits of 618 fM for IL-6 and 766 fM for insulin, demonstrating significant sensitivity improvements [73].

Graphene-based field-effect transistors (GFETs) have emerged as promising biosensors for real-time health monitoring, particularly due to their two-dimensional structure that supports flexibility and wearable applications. A cheap and flexible GFET was printed on a polyimide (Kapton) film with conductive ink printing for the detection of interleukin-6 (IL-6), an immune-related biomarker. The aptamers immobilized on the graphene surface guaranteed high selectivity for target analytes. The sensor also showed stable performance under bending (1.5–4.25 cm curvature) and allowed for the continuous detection of IL-6 within the concentration range of 10 pM–1 nM. Both points were further validated by another portable battery-powered miniaturized circuit board plugged as a free-standing wearable diagnostic tool [74].

**Figure 6:**
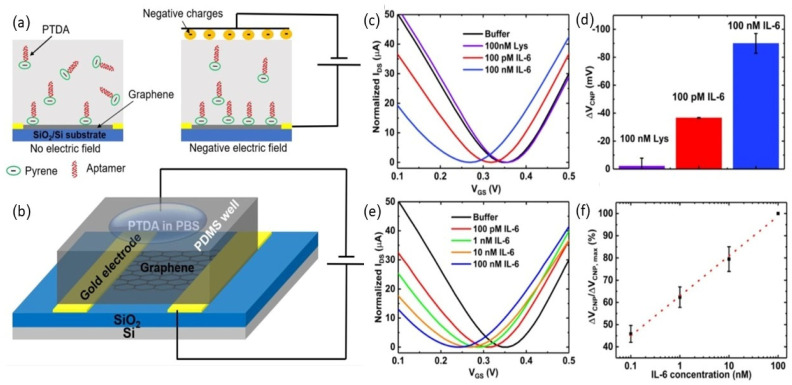
Fabrication and performance of flexible GFET aptasensor in terms of detecting IL-6. (**a**) Schematic of printed GFET device structure on polyimide substrate. (**b**) Functionalization scheme: Aptamer immobilization was achieved using PASE linkers functionalized on graphene surface. (**c**) Transfer curves evidencing device stability when bent. (**d**) Selectivity testing to exhibit response only for IL-6 rather than non-targeted proteins. (**e**) Shifts in electrical response depending on IL-6 concentration from 100 pM to 100 nM. (**f**) Calibration curve confirming detection limit of 100 pM [75].

A flexible GFET biosensor was fabricated using the conductive ink printing of graphene channels onto a polyimide (Kapton) film, chosen for its thermal stability and flexibility [75]. Figure 6a shows the schematic design of the flexible GFET on a polyimide substrate, while Figure 6b illustrates aptamer immobilization on the graphene surface, ensuring high molecular specificity. The biosensor demonstrated reliable electrical performance during mechanical deformation. Figure 6c depicts the stable responses towards repeated bending, and Figure 6d shows that IL-6 can be continuously detected from 10 pM to 100 nM. Moreover, a concentration-dependent shift in electrical responses from 100 pM to 100 nM of IL-6 was observed, as illustrated in Figure 6e. Figure 6f shows the corresponding calibration curve with a detection limit of 100 pM at pH 7.4. These results demonstrate the capabilities of printed GFET biosensors with a large area and high performance that can be achieved by their low-cost scalable synthesis, flexibility, and sensitivity.

Chronic obstructive pulmonary disease (COPD) constitutes a multifaceted disease of genetic, cellular, and molecular elements with pulmonary and extrapulmonary expressions in which systemic inflammation contributes to the rapid progression of the ailment, the development of comorbidities and the severity of morbidity and mortality [76,77,78]. The central inflammatory cytokine and cancer biomarker IL-6 is detectable in saliva, serum, and BALF for diagnostic and prognostic (initial relapse prediction and recurrence stage) purposes, as well as being a therapeutic target of chemopreventive drugs and monoclonal antibodies to immune checkpoint inhibitors [79]. In COPD, the levels of inflammatory cytokines such as IL-1β, IL-6, and TNFα are significantly elevated, with IL-6 serving as a key marker of systemic inflammation [80]. Multimarker models show better diagnostic performance, and IL-1β, IL-6, and TNFα are valuable markers for monitoring inflammatory phenotypes aiming to tailor COPD assessment. IL-6, an important inflammatory cytokine in COPD and other chronic diseases, is a significant prognostic factor. Sensitive IL-6 detection was witnessed using a label-free aptamer-modified GFET biosensor, prepared with graphene inkjet-printed from ultrasonic exfoliation. Functionalization used PBASE linkers to attach aptamers, and Au nanoparticles improved performance. The biosensor exhibited specific detection in PBS and was capable of detecting at 372 pM with potential application for non-invasive COPD biomarker monitoring [81].

Recently, an inkjet-printed graphene ink-based liquid-gated GFET biosensor that was jetted, sonicated–exfoliated and optimized for jetting was developed using aptamer functionalization. Functionalization with PBASE linkers allowed for aptamer immobilization for IL-6 specificity. Detection was based on the switching of I–V transfer curves, and selective sensing was achieved in PBS with a detection limit of 372 pM [82].

### 3.8. P24 Biomarker

HIV is a viral infection that causes the immune system to weaken over time, and if left untreated, it can develop into acquired immunodeficiency syndrome (AIDS). HIV infection is still a worldwide epidemic threatening human health, for which no effective treatment has been developed, and opportunistic infections exacerbate the condition of immunocompromised individuals [83,84]. Therefore, an early and accurate diagnosis is essential. HIV is a serious global public health challenge frequently associated with cardiovascular disease (CVD) and rheumatoid arthritis (RA). S. Islam et al. constructed an FET nanodevice based on amine-functionalized graphene covalently modified with antibodies (anti-p24, anti-cTn1, anti-CCP), facilitating the sensitive detection of biomarkers [27]. Upon the binding of the target antigen on the surface of graphene and subsequently coming in contact with its specific antibody, local doping inside the channel is reconfigured, which dynamically modulates resistance change as being directly proportional to analyte concentration. The device showed a linear respose from 1 fg/mL to 1 µg/mL, attaining detection limits of 100 fg/mL for p24 and 10 fg/mL for cTn1 and CCP.

Kim et al. fabricated a GFET on flexible substrates of polyethylene terephthalate, which could be used for the detection of attomolar levels of HIV-1. The antibodies were immobilized on the graphene gate by PBASE linkers, and virus binding resulted in a downward shift in Dirac point voltage due to electrostatic gating. It presented a remarkable LOD of 47.8 aM with excellent sensitivity [85].

J.W. Kim et al. reported a flexible graphene FET (GFET) based on liquid coplanar gate arrays on polyethylene terephthalate (PET) substrates for the ultrasensitive detection of HIV-1. Antibodies were anchored to graphene via PASE linkers in such a way that only specific antigen–antibody binding was possible. After virus attachment, electrostatic gating shifted the Dirac point downward, which allowed for highly sensitive detection. Amazingly, a GFET exhibited an ultralow LOD at the attomolar scale (47.8 aM) and a broad linear range from 47.8 aM to 10.55 nM. Notably, a saline environment did not destroy the sensor’s performance (Na^+^/Cl^−^) in solution, which indicated that it is a direct, low-cost wearable device for HIV detection [85].

Early HIV diagnosis is essential for reducing transmissions and for initiating effective antiretroviral therapy (ART), but traditional immunoassays are expensive and restricted to point-of-care (POC) settings. A nano-mechanical cantilever-based biosensor was fabricated with successful rapid screening for HIV-1 p24 at an ultralow detection limit of 100 fg/mL (in solution) and 1 pg/mL (serum) [86]. Functionalized with broadly cross-reactive mAbs, the system measured p24 from multiple HIV-1 subtypes, was compatible with blood pretreatment protocols and provided direct electronic readout.

### 3.9. Prostate-Specific Antigen (PSA) Biomarker

Prostate cancer, which accounts for 10% of all cancer deaths globally, requires early detection through protein biomarkers, for which graphene-based biosensors have gained increasing attention due to their unique physicochemical properties and developed diagnostics into an ultimately efficient framework for more sensitive patient diagnosis and care at large [87]. Field-effect transistors (FETs) based on graphene were proposed as an ideal platform for biosensing because they have a relatively high surface area, large carrier mobility and biocompatibility [88,89]. With femtomolar detection limits, graphene FETs have been widely applied to biomolecule identification, and recent studies highlight their effectiveness in detecting prostate-specific antigen (PSA) for prostate cancer diagnostics.

A flexible biosensor was fabricated using 3D hierarchical biocomposites of hollow pollen microcapsules coated with graphene and assembled on polyethylene terephthalate [90]. Antibody immobilization provided tunable specificity, enabling ultrasensitive PSA detection down to 1.7 × 10^−15^ M with real-time feedback. The device outperformed 2D graphene sensors and maintained stable performance under repeated bending conditions, highlighting its diagnostic potential.

Point-of-care (PoC) devices offer PSA detection in resource-limited settings, but many rely on labeled assays with poor sensitivity and complex workflows. Therefore, a label-free dielectrophoresis-assisted GFET on a Compact disk-based microfluidic platform was fabricated [91]. Based on the use of optimized coplanar gate electrodes, the sensor exhibited a detection limit of 1 pg/mL and a dynamic range extending to 4 ng/mL, as well as good selectivity, and proof of principle was given by measurements in human serum.

**Figure 7:**
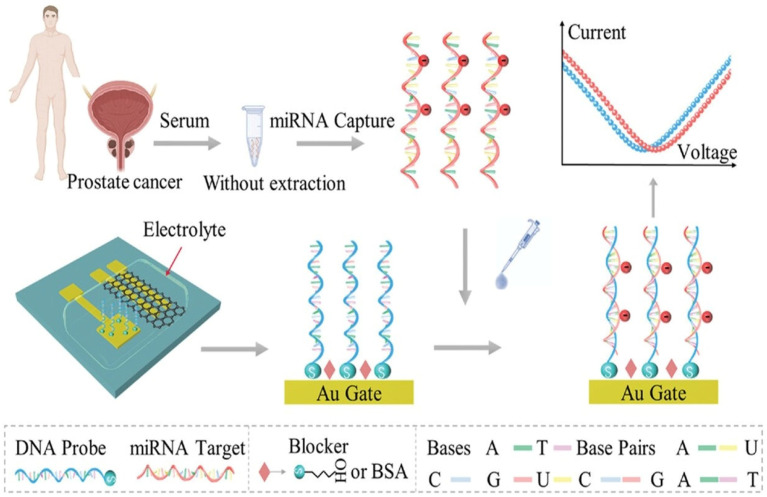
Schematic representation of graphene-based field-effect transistor biosensor, where functionalized gate electrodes capture target biomolecules and induce measurable Dirac voltage shifts for highly sensitive, label-free detection [93].

Prostate cancer ranks as one of the more common causes of cancer-related deaths in men, and PSA remains the best marker for its early detection. Traditional detection methods like immunoassays and chromatography have some disadvantages such as high cost, restricted portability, complex operation, etc. To overcome these limitations, Kong et al. reported a PSA-specific aptasensor based on a graphene field-effect transistor (GFET) with its surface modified by 1-pyrenebutyric acid N-hydroxysuccinimide ester (PBASE). The sensor demonstrated a large linear scope of 100 fM to 100 nM and an ultralow detection limit of 0.35 pM. Of significance was its high selectivity to soluble interferents and stability in human serum with recoveries of 97–100%, which underscored its prospect for clinical diagnostics as well as POC applications [92]. PSA continues to be the cornerstone biomarker for prostate cancer screening; however, the lack of specificity leads to inaccurate diagnostics. To overcome this, alternative markers such as microRNA-21 (miRNA-21) have been explored. M. Deng et al. reported an alcohol vapor sensor based on a solution-gated graphene transistor (SGGT) for the direct label-free detection of miRNA-21. In this setup, the DNA probes adsorbed on a Au gate were hybridized with miRNA-21, creating a well-defined Dirac voltage shift. The graphene channel, Au source/drain electrodes and DNA-functionalized gate facilitated acurate sensing, as depicted in Figure 7. The biosensor achieved an ultralow detection limit of 10^−20^ M, with a broad dynamic range from 10^−20^ to 10^−12^ M, offering significant potential to complement PSA in early prostate cancer diagnosis [93].

M. Deng et al. introduced a portable dual-gate solution-gated graphene transistor (SGGT) platform for the simultaneous detection of prostate cancer biomarkers prostate-specific antigen (PSA) and sarcosine (SAR). Single-layer CVD graphene serves as the channel, while two Au gate electrodes are functionalized with high-affinity aptamers (Apt-PSA-D and Apt-SAR). A pulsed electric field applied via a pulse electrode accelerates target–probe binding. Biomarker capture alters the electric double layer’s capacitance at the gate, producing Dirac point shifts; PSA binding decreases capacitance (positive ΔVDirac), whereas SAR binding increases it (negative ΔVDirac). The sensors yielded linear responses from 0.01 fg·mL^−1^ to 1 ng·mL^−1^, with limits of detection of 0.01 fg·mL^−1^ and detection times ≈ 4.5 min (PSA) and ≈13 min (SAR), and improved diagnostic accuracy versus PSA alone in clinical samples [28]. GFET nano-biosensors functionalized with engineered antibody fragments now enable the highly sensitive and multiplexed detection of Lyme disease antigens. By using single-chain variable fragment (scFv) antibodies positioned close to the graphene surface, the platform achieves pg mL^−1^ detection limits and a ~4000-fold sensitivity improvement over conventional GFETs. These advances provide early-stage, point-of-care diagnostic potential for Lyme disease and significantly reduce false positives through multiplexed antigen detection [94]. GFET biosensors now enable the ultrasensitive, rapid, and selective detection of disease biomarkers in biological fluids. Cortisol is a key example, as abnormal levels are linked to stress-related and metabolic disorders. Recent immuno-GFET designs with engineered antibodies and optimized graphene functionalization achieve very low detection limits, wide dynamic ranges, and reliable performance in real samples, highlighting the strong clinical potential of GFETs for real-time, non-invasive health monitoring and point-of-care diagnostics [95].

## 4. Comparative Perspective on GFET Biosensors and Alternative Sensing Technologies

Despite the fact that GFET biosensors exhibit ultralow detection limits on well-controlled laboratory-scale setups, benchmarking their performance with respect to other existing state-of-the-art sensing platforms is indispensable. Devices based on carbon nanotube (CNT) FETs and molybdenum disulfide (MoS_2_) possess an intrinsic bandgap that enables high on/off ratios, typically with lower carrier mobility compared to graphene. Label-free detection is also possible with CNT-FETs and MoS_2_-FETs. Silicon nanowire FETs are well established in fabrication processes and are demonstrated to enable a better reproducibility of device characteristics, though at the expense of a more complicated process as well as a high operating voltage [36].

Optical-based assays, including SPR and fluorescence, where specificity is desirable (e.g., molecular conjugation to the biomolecules is measured), have good sensing performance in terms of having been clinically validated beyond proof of principle but require bulky instrumentation and may not be ideal in terms of a portable or wearable form factor [36]. Electrochemical sensors are still clinically rooted and very robust in intricate biological environments, but they may rely on labeling strategies or redox mediators with relatively high power consumption.

In contrast, label-free real-time electrical detection could be achieved using GFET biosensors with good transconductance and compatibility with respect to flexible substrates. However, their performance in physiological environments is likely impacted by Debye screening effects, variability in surface chemistry and signal drift [36,96].

As the reported limits of detection (LODs) depend strongly on the specific analyte, assay configuration, and experimental conditions, the values listed below in Table 2 are representative examples rather than direct quantitative benchmarks.

## 5. Clinical Translation Prospects and Remaining Challenges

Although the analytical sensitivity of GFET biosensors is excellent in a laboratory setting, the eventual realization of their point-of-care (POC) diagnostic systems based on these sensors presents several fundamental and practical challenges [100,101,102,103]. A realistic consideration of these challenges is critical in shaping the future direction of GFET research.

Device-to-device variability is one of the main challenges to their clinical use. The synthesis conditions of graphene (e.g., CVD grain boundaries), transfer-induced defects, and inhomogeneity in surface functionalization cause nonuniform electrical properties between sensor arrays. Reproducible manufacturing protocols and quality control standards need to be adopted for regulatory approval and large-scale deployment. Developments in wafer-scale CVD graphene growth, automated transfer methods and batch-compatible biofunctionalization approaches are key to enhance device uniformity.

Operation in physiological-relevant fluids (serum, plasma, saliva) presents further challenges such as Debye screening, nonspecific adsorption and biofouling. These factors may deteriorate the signal-to-noise ratio and also introduce baseline drift, affecting long-term quantitative reliability. Antifouling surface chemistries, strategies to orient receptors optimally and differential or reference channel designs are alternatives with potential. Environmental stability can be further enhanced by encapsulation methods and microfluidic integration.

Unlike laboratory immunoassays, clinical diagnostics need reliable calibration stability and long-term reproducibility. Charge trapping-induced drift, environmental fluctuations and the instability of the interface are still the major limitations of GFET platforms. In future, the development of more effective passivation layers, stable linker chemistries and well-integrated on-chip referencing could further stabilize quantitative performance when configured with these optimized parameters.

In terms of clinical translation, scalability and adhering to regulatory standards (e.g., repeatability, robustness, validation in batches) are as important as sensitivity. Despite the compatibility of graphene with semiconductor manufacturing technologies, high-yield and low-cost fabrication under tight performance specifications has yet to be resolved. Standardized manufacturing procedures and validation in good manufacturing practice (GMP)-compatible conditions will be prerequisites for regulatory approval.

In spite of the abovementioned challenges, GFET-based biosensors exhibit unique characteristics, e.g., operation without any label, as well as fast on-chip electrical detection and low power consumption, in addition to mechanically flexible devices (available for wearable applications), which pave the way for decentralized diagnostic and wearable health monitoring. Instead of replacing established laboratory tests, GFETs will probably first become useful in clinical niche applications where real-time monitoring, portability or integration with wearables is required. Hence, future studies should focus on system-level integration and long-term stability in terms of real biological matrices, as well as multicenter validation trials in order to close the gap between proof-of-concept demonstrations and clinically implementable POC devices.

## 6. Conclusions

This review highlights the remarkable progress of graphene-based field-effect transistor (GFET) biosensors, which have emerged as a core element in current diagnostic research. Functionalized graphene interfaces integrated with advanced transduction architectures enabled GFETs to read out with extraordinary high detection sensitivity across various biomarkers such as clusterin, thrombin, cytokine interleukin-6 (IL-6), microRNAs, estrogen receptor α, Carcinoembryonic antigen, HepG2 exosomes, Polyclonal antibodies and antigens (P24) and prostate-specific antigen. These advances reveal not only the great analytical performance of graphene but also its compatibility with biological fluids such as serum, saliva and urine in terms of minimally invasive diagnosis. The integration of material development, electronic design, and biological assay is a barrier to using GFET biosensors in clinical applications and as a portable health monitor. Efforts should be made to explore the integration of multiple sensors, device miniaturization and validation with patient samples in order to translate laboratory discoveries into viable point-of-care healthcare devices.

## Figures and Tables

**Table 1:** GFET-based biomarkers along with their detectable diseases and limits of detection.

S. No.	Biomarker	Disease	Limit of Detection	Ref.
1	Clusterin	Alzheimer’s disease	300 fg/mL (4 fM)	[21]
2	Thrombin	Coagulation disorders/cardiovascular complication	2.6 pM	[22]
3	Estrogen receptor α	Breast cancer	2.62 fM	[23]
4	Nano-denatured bovine serum albumin (nano-dBSA)	Cancer	337.58 fg/mL	[24]
5	MicroRNA (mi-RNA)	Cancer/gene-related diseases	10 fM	[17]
6	Hepatoma exosome	Liver cancer	242 particles/mL	[25]
7	Cytokine IL-6	Non-invasive saliva diagnosis	12.2 pM	[26]
8	Polyclonal antibodies and antigens (P24)	Human immunodeficiency virus (HIV)	100 fg/mL	[27]
9	Prostate-specific antigen	Prostate cancer	0.01 fg/mL	[28]

**Table 2:** Comparison of GFET biosensors with other sensing materials.

S. No.	Material	Limit of Detection	Ref.
1	MoS_2_ FET	5.98 × 10^−5^ nM	[97]
2	Carbon nanotubes	6 particles/mL	[53]
3	Silicone nanowire bio-FET	2159 particles/mL	[98]
4	Molecularly imprinted polymer-based electrochemical material	1.5 U m/L	[99]
5	GFET	242 particles/mL	[25]

## Data Availability

No new data were created or analyzed in this study. Data sharing is not applicable to this article.
